# Aspects of the Neurospora crassa Sulfur Starvation Response Are Revealed by Transcriptional Profiling and DNA Affinity Purification Sequencing

**DOI:** 10.1128/mSphere.00564-21

**Published:** 2021-09-15

**Authors:** Lori B. Huberman, Vincent W. Wu, Juna Lee, Chris Daum, Ronan C. O’Malley, N. Louise Glass

**Affiliations:** a Plant and Microbial Biology Department, University of California, Berkeley, California, USA; b Energy Biosciences Institute, University of California, Berkeley, California, USA; c Plant Pathology and Plant-Microbe Biology Section, School of Integrative Plant Science, Cornell University, Ithaca, New York, USA; d U.S. Department of Energy Joint Genome Institute, Lawrence Berkeley National Laboratory, Berkeley, California, USA; e Environmental Genomics and Systems Biology Division, Lawrence Berkeley National Laboratory, Berkeley, California, USA; University of Georgia

**Keywords:** DAP-seq, nutrient sensing, sulfur starvation response, transcriptional profiling

## Abstract

Accurate nutrient sensing is important for rapid fungal growth and exploitation of available resources. Sulfur is an important nutrient source found in a number of biological macromolecules, including proteins and lipids. The model filamentous fungus Neurospora crassa is capable of utilizing sulfur found in a variety of sources from amino acids to sulfate. During sulfur starvation, the transcription factor CYS-3 is responsible for upregulation of genes involved in sulfur uptake and assimilation. Using a combination of RNA sequencing and DNA affinity purification sequencing, we performed a global survey of the N. crassa sulfur starvation response and the role of CYS-3 in regulating sulfur-responsive genes. The CYS-3 transcription factor bound the promoters and regulated genes involved in sulfur metabolism. Additionally, CYS-3 directly activated the expression of a number of uncharacterized transporter genes, suggesting that regulation of sulfur import is an important aspect of regulation by CYS-3. CYS-3 also directly regulated the expression of genes involved in mitochondrial electron transfer. During sulfur starvation, genes involved in nitrogen metabolism, such as amino acid and nucleic acid metabolic pathways, along with genes encoding proteases and nucleases that are necessary for scavenging nitrogen, were activated. Sulfur starvation also caused changes in the expression of genes involved in carbohydrate metabolism, such as those encoding glycosyl hydrolases. Thus, our data suggest a connection between sulfur metabolism and other aspects of cellular metabolism.

**IMPORTANCE** Identification of nutrients present in the environment is a challenge common to all organisms. Sulfur is an important nutrient source found in proteins, lipids, and electron carriers that are required for the survival of filamentous fungi such as Neurospora crassa. Here, we transcriptionally profiled the response of N. crassa to characterize the global response to sulfur starvation. We also used DNA affinity purification sequencing to identify the direct downstream targets of the transcription factor responsible for regulating genes involved in sulfur uptake and assimilation. Along with genes involved in sulfur metabolism, this transcription factor regulated a number of uncharacterized transporter genes and genes involved in mitochondrial electron transfer. Our data also suggest a connection between sulfur, nitrogen, and carbon metabolism, indicating that the regulation of a number of metabolic pathways is intertwined.

## INTRODUCTION

In nature, fungi must efficiently exploit nutrients to establish colonies and outcompete neighboring microbes. Along with essential nutrients such as carbon, nitrogen, and phosphate, sulfur is also required. In humans, mutations in sulfur acquisition and metabolism genes cause skeletal abnormalities and other serious medical conditions (1, 2). Pathogenic fungi unable to properly regulate sulfur utilization genes show reduced virulence (3).

Fungi acquire sulfur from a variety of sulfur sources. The organic sulfur substrates cysteine and methionine are preferred as they require minimal metabolic processing for use in cellular processes, such as translation and the use of *S*-adenosylmethionine (SAM) in methyl group transfer to various substrates. However, fungi also acquire sulfur from other organic and inorganic sources, including taurine, cysteic acid, sulfate, and aromatic sulfate compounds (4).

In the filamentous fungus Neurospora crassa, sulfur-responsive genes are regulated by the basic leucine zipper transcription factor CYS-3 (NCU03536) (5). During sulfur limitation, CYS-3 activates the expression of itself, genes encoding sulfur transporters, and enzymes required for sulfur metabolism (6). When sulfur is abundant, the F-box protein SCON-2 (NCU08563) and the Skp protein SCON-3 (NCU08991) are thought to act in an Skp, cullin, F-box (SCF) E3 ubiquitin ligase complex to degrade CYS-3 (7, 8). The reduced abundance of CYS-3 reduces expression of sulfur regulated genes.

A similar regulatory scheme exists in other filamentous fungi. A CYS-3 homolog, MetR, is required for utilization of nonpreferred sulfur sources in Aspergillus nidulans and Aspergillus fumigatus (3, 9). Like in N. crassa, an SCF E3 ubiquitin ligase complex is thought to regulate abundance of MetR in A. nidulans (10, 11). However, the genomes of A. nidulans and other fungi in the *Eurotiales* also contain a *metR* paralog, *metZ*. In A. nidulans, *metZ* is regulated by MetR; MetZ is involved in activating some sulfur starvation response genes (12).

Substantial work in N. crassa and other fungi elucidated aspects of sulfur regulation using classical and molecular genetics. However, global regulation of sulfur-responsive genes is not understood. We addressed this question by profiling the transcriptional response of N. crassa to sulfur starvation. To further characterize the role of CYS-3 in sulfur regulation, we combined RNA sequencing (RNA-seq) of a *cys-3* mutant with DNA affinity purification sequencing (DAP-seq) of the CYS-3 protein, allowing us to identify direct targets of the CYS-3 transcription factor. Along with genes involved in sulfur metabolism, transcription of a substantial number of genes encoding known sulfur transporters and uncharacterized major facility superfamily (MFS) transporters was directly regulated by CYS-3. Additionally, our data suggest a connection between sulfur, nitrogen, and carbon metabolism.

## RESULTS

### Genes encoding enzymes involved in sulfur acquisition and assimilation are regulated in response to sulfur starvation.

Sulfur is required for cell growth. To ensure sufficient sulfur is available, during sulfur limitation N. crassa cells upregulate expression of sulfur transporters and enzymes involved in sulfur metabolism (13–16). However, the global sulfur starvation response has not been elucidated. We therefore profiled the transcriptional response of N. crassa during sulfur starvation. We grew wild-type cells in media containing 800 μM sulfate for 24 h and washed the mycelia either in media lacking sulfur or containing 24 μM sulfate. We then exposed cells to sulfur starvation or 24 μM sulfate for 4 h prior to harvesting cells for RNA-seq. We chose 24 μM sulfate for expression profiling at 4 h because it repressed expression of known sulfur-responsive genes (13, 16) (see Fig. S1A and B in the supplemental material)—similar to exposure to Vogel’s minimal medium (VMM; 800 μM sulfate) (17–19)—and also supported growth of N. crassa (Fig. S1C).

10.1128/mSphere.00564-21.2 FIG S1 Sulfur-responsive genes are activated by CYS-3 in response to sulfur starvation. Shown are FPKM of (A) *ars-1* (NCU06041) and (B) *cys-14* (NCU04433) in wild-type and *cys-3^−^* cells exposed to 24 μM sulfate and sulfur starvation. Bars indicate the average of 3 biological replicates. Dots indicate individual data points. Average FPKM and standard deviation are indicated above or within each bar. (C) Wild-type cells were inoculated onto slants containing either VMM with 800 μM sulfate or VMM with 24 μM sulfate and incubated for 2 days at 30°C in constant dark and 4 days at 25°C in constant light. A representative image of at least 3 biological replicates is shown. Download FIG S1, TIF file, 1.1 MB. Copyright © 2021 Huberman et al. 2021 Huberman et al. https://creativecommons.org/licenses/by/4.0/ This content is distributed under the terms of the Creative Commons Attribution 4.0 International license.

**FIG 1:**
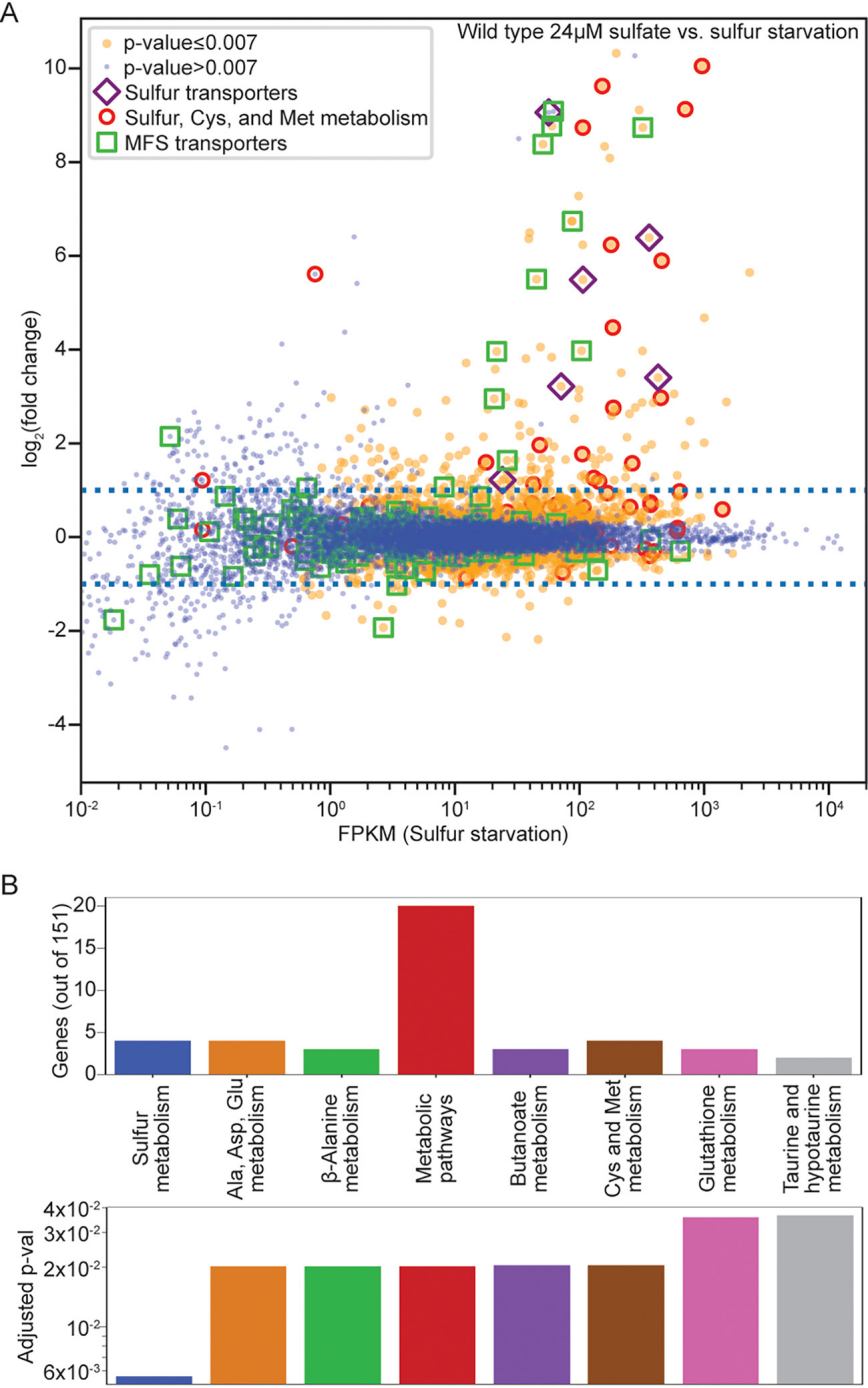
Genes encoding transporters and enzymes involved in sulfur metabolism are upregulated in response to sulfur starvation. (A) Differential expression analysis of wild-type cells exposed to 24 μM sulfate relative to media lacking sulfur. Predicted sulfur transporters are indicated by purple diamonds. Genes predicted to be involved in sulfur, cysteine, or methionine metabolism are indicated by red circles. Predicted MFS transporters are indicated by green squares. Dotted blue lines indicate a 2-fold change in expression. (B) Distribution of genes in KEGG categories (50) and adjusted *P* values reflecting the significance of enrichment of genes assigned to a given KEGG category, as calculated using FungiFun 2.2.8 (49), of the 151 genes at least 2-fold differentially expressed between wild-type cells exposed to 24 μM sulfate and media lacking sulfur.

Expression of 151 genes was at least 2-fold differentially regulated in cells exposed to sulfur starvation compared to cells exposed to 24 μM sulfate (Fig. 1A; see Fig. S2 and Data Set S1 in the supplemental material). Functional enrichment analysis of these 151 genes showed an overrepresentation of genes involved in sulfur utilization, including sulfur, cysteine, methionine, glutathione, taurine, and hypotaurine metabolism. These 151 genes also showed enrichment for alanine, aspartate, glutamate, β-alanine, and butanoate metabolism (Fig. 1B).

10.1128/mSphere.00564-21.3 FIG S2 Genes regulated in response to sulfur starvation in wild-type cells. A heat map of the expression level of the 151 genes that were at least 2-fold differentially expressed between wild-type cells exposed to 24 μM sulfate and sulfur starvation is shown. The upper heat map contains genes activated by sulfur starvation. The lower heat map contains genes repressed by sulfur starvation. Purple bars indicate genes whose promoters were bound by CYS-3 and were at least 2-fold differentially expressed between wild-type and *cys-3^−^* cells exposed to sulfur starvation. Download FIG S2, TIF file, 2.7 MB. Copyright © 2021 Huberman et al. 2021 Huberman et al. https://creativecommons.org/licenses/by/4.0/ This content is distributed under the terms of the Creative Commons Attribution 4.0 International license.

10.1128/mSphere.00564-21.9 DATA SET S1 Processed RNA-seq data. Normalized FPKM and differential expression analysis of wild type and *cys-3^−^* cells exposed to 24 μM sulfate and sulfur starvation. Download Data Set S1, XLSX file, 1.2 MB. Copyright © 2021 Huberman et al. 2021 Huberman et al. https://creativecommons.org/licenses/by/4.0/ This content is distributed under the terms of the Creative Commons Attribution 4.0 International license.

**FIG 2:**
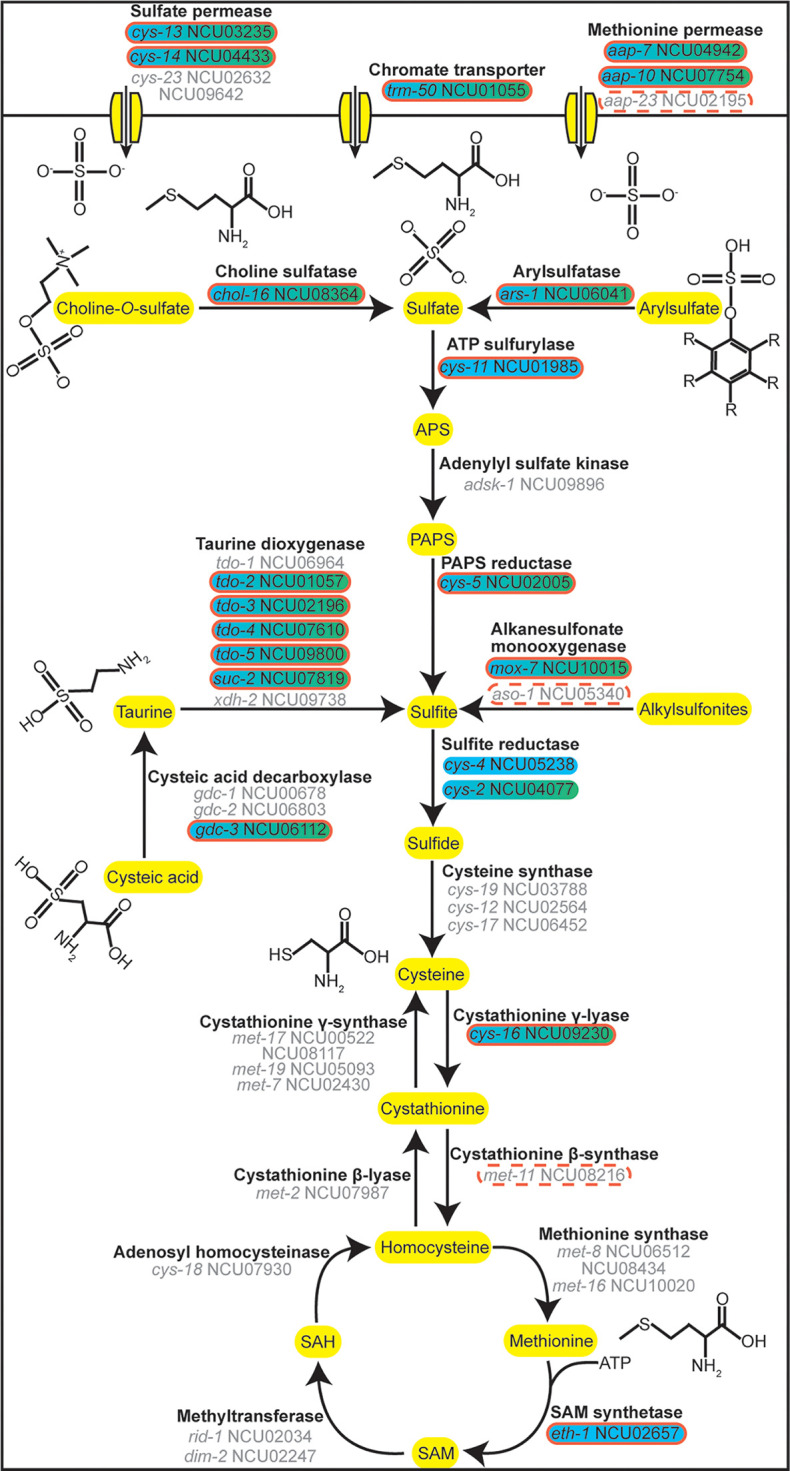
Genes involved in sulfur acquisition but not cysteine and methionine metabolism are regulated by CYS-3 in response to sulfur starvation. Shown is a cartoon of sulfur assimilation in N. crassa. Green shading indicates that the gene was differentially expressed by at least 2-fold in wild-type cells exposed to 24 μM sulfate compared to sulfur starvation. All of these differentially expressed genes were upregulated during sulfur starvation compared to 24 μM sulfate. Blue shading indicates that the gene was differentially expressed by at least 2-fold in wild-type compared to *cys-3^−^* cells exposed to sulfur starvation. All of these differentially expressed genes were activated by CYS-3 during exposure to sulfur starvation. Gray text indicates that the gene was not differentially expressed by at least 2-fold under either of the conditions. A solid red outline indicates that CYS-3 bound the gene’s promoter and that the gene was at least 2-fold differentially expressed in wild-type compared to *cys-3^−^* cells exposed to sulfur starvation. A dotted red outline indicates that CYS-3 bound the gene’s promoter in the unfiltered DAP-seq data but that the gene was not at least 2-fold differentially expressed under the tested conditions. There was a lower confidence that these promoters were bound by CYS-3 *in vivo* than promoters of genes with a solid red outline. APS, adenosine 5′-phosphosulfate; PAPS, 3′-phosphoadenosine-5′-phosphosulfate; SAM, *S*-adenosylmethionine; SAH, *S*-adenosylhomocysteine.

Expression of only 15 of the 151 differentially expressed genes was downregulated in response to sulfur starvation (Fig. 1A; Fig. S2). These 15 genes encoded proteins involved in the fungal cell wall, stress response, carbohydrate metabolism, and energy generation (Data Set S1). The other 136 differentially expressed genes were upregulated in response to sulfur starvation (Fig. 1A; Fig. S2). These 136 genes included genes encoding known and predicted enzymes involved in sulfur acquisition and metabolism (Fig. 2; Data Set S1).

N. crassa can utilize a number of compounds as sulfur sources. Preferred sulfur sources include cysteine and methionine. Nonpreferred sulfur sources, such as sulfate, sulfite, arylsulfate, and taurine, are converted into cysteine and methionine before use (4). Because genes involved in conversion of sulfate to cysteine and methionine were presumably necessary during exposure to 24 μM sulfate, we did not expect many genes involved in cysteine and methionine metabolism to be differentially expressed between 24 μM sulfate and media lacking sulfur. To our surprise, four genes in the cysteine and methionine metabolism Kyoto Encyclopedia of Genes and Genomes (KEGG) pathway and two additional genes with roles in cysteine and methionine metabolism were at least 2-fold more highly expressed during exposure to sulfur starvation than 24 μM sulfate (see Fig. S3 and Data Set S1 in the supplemental material). However, only the cystathionine γ-lyase gene *cys-16* (NCU09230), previously shown to be upregulated during sulfur starvation, is thought to be directly involved in the enzymatic pathway converting sulfate into cysteine and methionine (20, 21) (Fig. 2; Fig. S3).

10.1128/mSphere.00564-21.4 FIG S3 Genes involved in sulfur acquisition but not cysteine and methionine metabolism are directly regulated by CYS-3 in response to sulfur starvation. A heat map of the expression level of genes involved in sulfur acquisition and metabolism is shown. Purple bars indicate genes whose promoters were bound by CYS-3 and were at least 2-fold differentially expressed between wild-type and *cys-3^−^* cells exposed to sulfur starvation. Download FIG S3, EPS file, 1.7 MB. Copyright © 2021 Huberman et al. 2021 Huberman et al. https://creativecommons.org/licenses/by/4.0/ This content is distributed under the terms of the Creative Commons Attribution 4.0 International license.

Close examination of genes involved in conversion of sulfate to adenosine 5′-phosphosulfate (APS) to 3′-phosphoadenosine-5′-phosphosulfate (PAPS) to sulfite to sulfide, showed expression of most of these genes increased only slightly in response to sulfur starvation (20). In fact, of the 5 genes involved in this metabolic pathway, expression of only the PAPS reductase gene *cys-5* (NCU02005) and sulfite reductase gene *cys-2* (NCU04077) increased by more than 2-fold in response to sulfur starvation (Fig. 2; Fig. S3 and Data Set S1).

As expected, a much larger increase in expression was seen in genes involved in metabolism of sulfur sources that feed into the pathway converting sulfate to sulfide, including those involved in metabolism of alkylsulfonites, arylsulfate, choline-*O*-sulfate, cysteic acid, and taurine (16, 22, 23). Expression of arylsulfatase (*ars-1* [NCU06041]), which converts arylsulfate to sulfate, increased 427-fold. Similarly, choline sulfatase (*chol-16* [NCU08364]), which converts choline-*O*-sulfate to sulfate, was 22-fold upregulated. Five genes encoding taurine dioxygenases, responsible for converting taurine to sulfite (*tdo-2* [NCU01057], *tdo-3* [NCU02196], *tdo-4* [NCU07610], *tdo-5* [NCU09800], and *suc-2* [NCU07819]) increased more than 59-fold. Expression of 2 additional predicted taurine dioxygenase genes, *tdo-1* (NCU06964) and *xdh-2* (NCU09738), was unchanged (Fig. 2; Fig. S3 and Data Set S1). Cysteic acid can also be used as a sulfur source, but it must first be converted to taurine (24). Three genes in the N. crassa genome are predicted to encode cysteic acid decarboxylases, which mediate this conversion. However, expression of only *gdc-3* (NCU06112) increased more than 2-fold in response to sulfur starvation (Fig. 2; Fig. S3 and Data Set S1). Alkanesulfonate monooxygenase converts alkylsulfonites to sulfite. Although 2 genes in the N. crassa genome are predicted to encode alkylsulfonate monooxygenases, *mox-7* (NCU10015) was 7-fold upregulated in response to sulfur starvation, while *aso-1* (NCU05340) was barely expressed during either sulfur starvation or exposure to 24 μM sulfate (Fig. 2; Fig. S3 and Data Set S1).

Glutathione is a sulfur-containing antioxidant synthesized from glutamate and cysteine. Several glutathione transferases (*gst-2* [NCU04109] and *gst-6* [NCU00549]) and the glutamate-cysteine ligase catalytic subunit (*gcl-1* [NCU01157]) were also upregulated during sulfur starvation (Data Set S1).

### Sulfur starvation results in activation of genes involved in nitrogen and carbon metabolism.

While expression of many genes involved in converting sulfate to cysteine and methionine was generally unchanged in response to sulfur starvation, expression of genes involved in converting other nonpreferred sulfur sources to sulfate or sulfite was significantly upregulated (Fig. 2). We hypothesized genes encoding transporters and secreted enzymes involved in liberating sulfur from insoluble sources might also be upregulated during sulfur limitation.

Experiments using classical and molecular genetics showed expression of sulfur transporters increases under sulfur limitation (13, 15). As expected, expression of the sulfate permease gene *cys-14* (NCU04433) increased by 84-fold. Although the sulfate permease gene *cys-13* (NCU03235) is mainly expressed in conidia (25), *cys-13* expression also increased by 2-fold (Fig. 2; Fig. S3 and Data Set S1). Both sulfur and chromate, a sulfate analog, are transported into the cell via sulfate permeases (26). Expression of the chromate transporter gene *trm-50* (NCU01055) increased 45-fold during sulfur starvation, suggesting *trm-50* may also play a role in sulfur transport (27) (Fig. 2; Fig. S3 and Data Set S1). Expression of two genes encoding predicted methionine permeases, *aap-7* (NCU04942) and *aap-10* (NCU07754), also increased over 9-fold during sulfur starvation (Fig. 2; Fig. S3 and Data Set S1). Additionally, expression of two putative peptide transporter genes, *mfs-9* (NCU05079) and *opt-3* (NCU07894), and two predicted amino acid permease genes, *aap-18* (NCU08880) and *aap-22* (NCU04435), increased more than 5-fold (Data Set S1). However, expression of predicted sulfate transporter genes *cys-23* (NCU02632) and NCU09642, as well as the putative methionine permease gene *aap-23* (NCU02195), did not change significantly during sulfur starvation (Fig. 2; Fig. S3 and Data Set S1).

The substantial induction of sulfur transporter gene expression led us to hypothesize expression of other transporter genes may also increase during sulfur starvation. Indeed, expression of 10 MFS transporters increased at least 2-fold during sulfur starvation (Fig. 1A). We also observed upregulation of iron-sulfur clusters transporter gene *fes-4* (NCU05029), multidrug transporter gene *abc-9* (NCU03776), P-type ATPase gene *ph7* (NCU08147), voltage-gated potassium channel gene *trm-68* (NCU2887), and urea transporter gene *urt* (NCU09909) (Data Set S1).

Because cysteine and methionine are preferred sulfur sources, N. crassa and other fungi can utilize proteins as a sulfur source obtained by secreting extracellular proteases during sulfur starvation (14, 28–30). A global search for protease genes upregulated during sulfur starvation identified 15 protease genes, including *spr-7* (NCU07159) whose expression increased 50-fold during sulfur starvation (Data Set S1).

Some fungal proteases are regulated by sulfur, nitrogen, and carbon starvation, leading to the hypothesis that sulfur metabolism may be connected with nitrogen and carbon metabolism (14, 29). Indeed, genes involved in nitrogen metabolism, in particular, those involved in amino acid metabolism, were activated during sulfur starvation. These genes included the aminotransferase gene *glu-2* (NCU08998), predicted aminotransferase gene NCU08011, and kynurenine-oxoglutarate transaminase gene *nic-4* (NCU03347) (Data Set S1). Expression of the formamidase gene *fma-1* (NCU02361) also increased. Additionally, we observed induction of genes involved in nucleic acid uptake and utilization, including nuclease gene *nuc-14* (NCU09788) and predicted extracellular DNase gene NCU09525, as well as 5′-nucleotidase gene *nut-1* (NCU09659), uracil-5-carboxylate decarboxylase gene *uc-7* (NCU06417), uracil phosphoribosyltransferase gene *uc-8* (NCU06261), and guanine deaminase gene *gua-5* (NCU07309) (Data Set S1).

Genes related to carbon metabolism were also upregulated during sulfur starvation, including glycosyl hydrolase genes *gh5-5* (NCU05882), *gh125-2* (NCU8371), *gh71-5* (NCU07355), and *gh61-2* (NCU07760), as well as cellobiose dehydrogenase-like protein gene NCU08432, which when deleted, improves growth on pectin (31) (Data Set S1). We also saw upregulation of NCU09519, which is involved in ascorbic acid metabolism, and pyridoxal reductase gene *pdx-6* (NCU07402), which is involved in vitamin B_6_ metabolism (Data Set S1). Expression of genes encoding proteins involved in oxidation-reduction reactions and electron transfer also increased (Data Set S1).

### CYS-3 activates many genes involved in the response to sulfur starvation.

The transcription factor CYS-3 is required for expression of sulfur-regulated genes, and during sulfur starvation, expression of *cys-3* is upregulated (5). Our data confirmed *cys-3* upregulation during sulfur starvation (Fig. S3 and Data Set S1). The F-box protein SCON-2 is a member of an SCF E3 ubiquitin ligase complex responsible for CYS-3 degradation (7, 8); expression of *scon-2* also increases during sulfur starvation (32), which our data confirmed. Expression of the Skp1 homolog gene *scon-3*, thought to work with SCON-2 to regulate CYS-3 protein levels, was unchanged, as previously reported (8) (Fig. S3 and Data Set S1).

To further investigate genes regulated by CYS-3, we isolated a homokaryotic *cys-3^−^* strain from the heterokaryotic *cys-3* mutant in the N. crassa deletion collection (33). Previous work done on *cys-3* used a partial *cys-3* deletion (6, 34); based on sequence and restriction site data, this *cys-3* mutant is missing part of the basic leucine zipper domain in exon 2 (see Fig. S4A in the supplemental material) (6). In the deletion collection *cys-3* mutant, a hygromycin resistance cassette replaced the region from 447 bp upstream of the *cys-3* start codon to 949 bp into the *cys-3* open reading frame. This region included a portion of the *cys-3* 5′ untranslated region, the first 301 bases of the *cys-3* coding region, and a portion of the first *cys-3* intron (*cys-3^−^*) (Fig. S4A and B) (33). Like other *cys-3* mutants, this *cys-3* partial deletion strain was unable to grow without methionine supplementation and was unable to activate expression of known sulfur-responsive genes during exposure to sulfur starvation conditions (Fig. S1 and S4C) (6).

10.1128/mSphere.00564-21.5 FIG S4 The partial deletion of *cys-3* is unable to grow without methionine supplementation. (A) Cartoon indicating the region of the *cys-3* gene that was replaced with a hygromycin resistance cassette in the *cys-3^−^* mutant in yellow. The base pair coordinates below the cartoon start with 0 at the *cys-3* start codon. The activating transcription factor domain (PTHR13044) (E value = 2.7 × 10^−55^), which spans amino acids 40 to 287, is shown in light blue. The basic leucine zipper domain (PF07716) (E value = 6.7 × 10^−13^), which includes the DNA binding domain and spans amino acids 197 to 248, is shown in pink. (B) Plot of RNA sequencing alignment density of *cys-3* in wild-type and *cys-3^−^* cells exposed to either 24 μM sulfate or sulfur starvation made using Integrative Genomics Viewer (J. T. Robinson, H. Thorvaldsdóttir, W. Winckler, M. Guttman, E. S. Lander, G. Getz, and J. P. Mesirov, Nat Biotechnol 29:24–26, 2011, https://doi.org/10.1038/nbt.1754). The RNA sequencing alignment density is lined up with the regions of the cartoon shown in panel A. The blue line in the *cys-3^−^* alignments indicates a T-to-C mutation at base 1147 in the intron of the *cys-3* gene. (C) Wild-type and *cys-3^−^* cells were inoculated onto slants containing either VMM or VMM plus 500 mg/liter methionine and incubated for 2 days at 30°C in constant dark and 4 days at 25°C in constant light. A representative image of at least 3 biological replicates is shown. Download FIG S4, TIF file, 2.1 MB. Copyright © 2021 Huberman et al. 2021 Huberman et al. https://creativecommons.org/licenses/by/4.0/ This content is distributed under the terms of the Creative Commons Attribution 4.0 International license.

**FIG 3:**
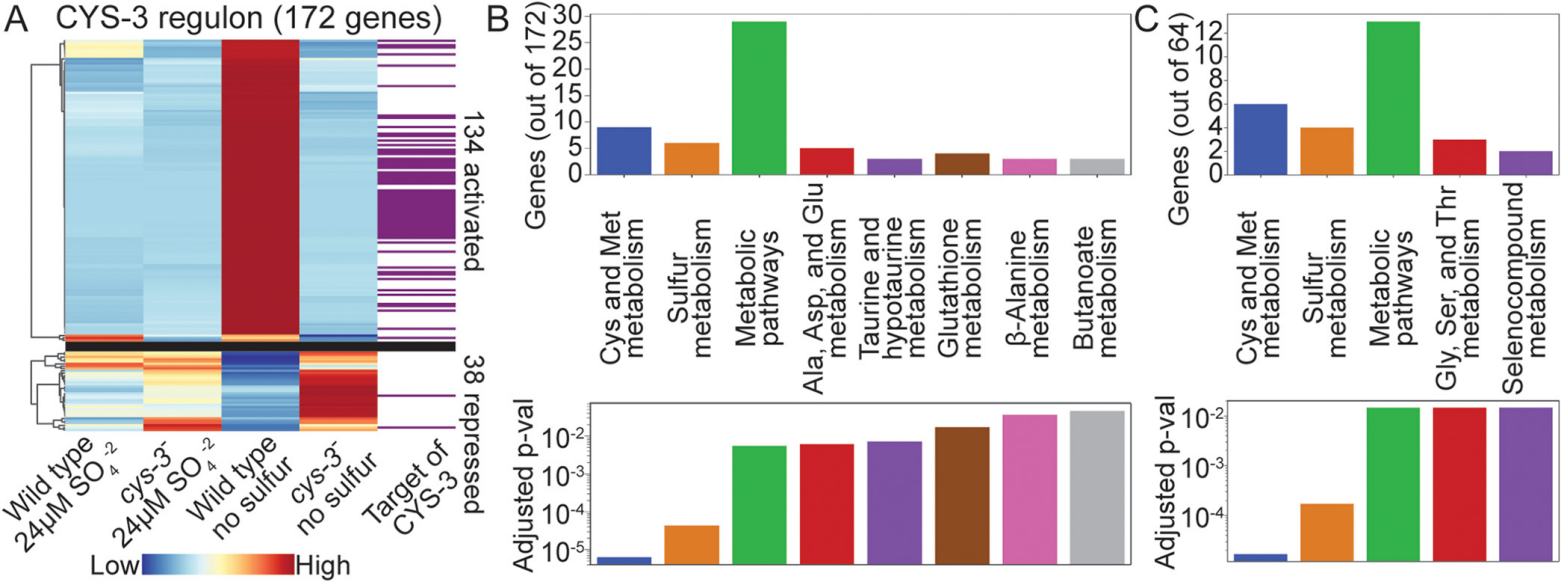
CYS-3 binds the promoters and regulates the expression of genes involved in amino acid and sulfur metabolism. (A) Heat map of the expression level of the 172 genes that were at least 2-fold differentially expressed between wild-type and *cys-3^−^* cells exposed to sulfur starvation. The upper heat map contains genes activated by CYS-3. The lower heat map contains genes repressed by CYS-3. Purple bars indicate genes whose promoters were bound by CYS-3 using DAP-seq. (B) Distribution of genes in KEGG categories (50) and adjusted *P* values reflecting the significance of enrichment of genes assigned to a given KEGG category, as calculated using FungiFun 2.2.8 (49), of the 172 genes that were at least 2-fold differentially expressed between wild-type and *cys-3^−^* cells exposed to sulfur starvation. (C) Distribution of genes in KEGG categories (50) and adjusted *P* values reflecting the significance of enrichment of genes assigned to a given KEGG category, as calculated using FungiFun 2.2.8 (49), of the 64 genes whose promoters were bound by CYS-3 and that were at least 2-fold differentially expressed between wild-type and *cys-3^−^* cells exposed to sulfur starvation.

We exposed this *cys-3^−^* mutant to sulfur starvation for 4 h and measured the transcriptome using RNA-seq. The remaining portion of the *cys-3* gene was transcribed in the mutant but was not upregulated during sulfur starvation (Fig. S3 and S4B and Data Set S1). Since *cys-3* activates its own expression (6, 35), lack of induction of the remaining portion of *cys-3* was consistent with a lack of CYS-3 activity. Expression of 172 genes was at least 2-fold differentially expressed between *cys-3^−^* and wild-type cells (Fig. 3A; see Fig. S5 in the supplemental material). Functional enrichment of these 172 genes showed overrepresentation of the same categories that were enriched in the set of genes differentially expressed in wild-type cells exposed to sulfur starvation compared to 24 μM sulfate: sulfur, cysteine, methionine, glutathione, taurine, hypotaurine, alanine, aspartate, glutamate, β-alanine, and butanoate metabolism (Fig. 3B).

10.1128/mSphere.00564-21.6 FIG S5 CYS-3 regulates sulfur transporters, MFS transporters, and genes involved in sulfur, cysteine, and methionine metabolism. Shown is differential expression analysis of *cys-3^−^* cells relative to wild-type cells after a shift to sulfur starvation. Purple diamonds indicate predicted sulfur transporters. Red circles indicate genes predicted to be involved in sulfur, cysteine, or methionine metabolism. Blue pentagons indicate predicted MFS transporters. Green squares indicate genes whose promoters were bound by CYS-3. Dotted blue lines indicate a 2-fold change in expression. Download FIG S5, TIF file, 0.8 MB. Copyright © 2021 Huberman et al. 2021 Huberman et al. https://creativecommons.org/licenses/by/4.0/ This content is distributed under the terms of the Creative Commons Attribution 4.0 International license.

We further analyzed similarities between genes differentially expressed during sulfur starvation in wild-type cells and genes regulated by CYS-3. Of the 172 genes differentially expressed by at least 2-fold in *cys-3^−^* cells compared to wild-type cells, 119 were also at least 2-fold differentially expressed in wild-type cells exposed to sulfur starvation (Fig. 3A; Data Set S1).

CYS-3 functions as a transcriptional activator (6, 35, 36). Transcriptional profiling showed expression of 134 genes decreased by at least 2-fold in *cys-3^−^* cells. Comparison of these 134 genes to the genes differentially expressed during sulfur starvation showed 114 of these 134 genes were upregulated during sulfur starvation in wild-type cells (Fig. 3A; Data Set S1). In comparison, only 38 genes were upregulated by at least 2-fold in *cys-3^−^* cells compared to wild-type cells. Of these 38 genes, only 5 were downregulated by at least 2-fold during sulfur starvation in wild-type cells (Fig. 3A; Data Set S1).

Functional enrichment analysis showed CYS-3-regulated genes were involved in cysteine and methionine metabolism (Fig. 3B). Because sulfate is converted to cysteine and methionine as part of sulfur metabolism, CYS-3 might also regulate these genes when the cell is exposed to sulfate. Eleven genes were at least 2-fold differentially expressed between wild-type and *cys-3^−^* cells during exposure to 24 μM sulfate; 7 of these 11 genes were downregulated by at least 2-fold in the *cys-3^−^* mutant. These 7 genes included 2 predicted sulfur transporter genes, *cys-13* and *aap-7*, and 4 genes involved in sulfur metabolism, *cys-5* (NCU02005), *cys-2*, *cys-11* (NCU01985), and *gst-4* (NCU10521) (Data Set S1). Only 4 genes were upregulated in *cys-3^−^* cells exposed to 24 μM sulfate: *cys-22* (NCU06625), *eas* (NCU08457), NCU16018, and NCU16023 (Data Set S1). These data support previous studies showing CYS-3 acts as a transcriptional activator during sulfur limitation.

### CYS-3 directly regulates genes associated with sulfur import, assimilation, and metabolism.

Genes differentially expressed in the *cys-3^−^* mutant compared to the wild type could be either directly or indirectly regulated by CYS-3. We therefore identified promoters bound by CYS-3 using DNA sequencing to identify regions of genomic DNA bound by *in vitro* transcribed and translated CYS-3, a technique known as DAP-seq (37). We identified 643 genes with 669 CYS-3 DNA binding sites within 3,000 bp upstream of their translational start sites (see Data Set S2 in the supplemental material). Because DAP-seq is an *in vitro* method of identifying DNA binding sites, it is possible CYS-3 did not regulate expression of all 643 genes *in vivo* (37). We therefore filtered our DAP-seq data to identify genes whose promoters were bound and whose expression was regulated by CYS-3 by comparing CYS-3 DAP-seq data to RNA-seq data of the *cys-3^−^* mutant.

10.1128/mSphere.00564-21.10 DATA SET S2 Processed DAP-seq data. DAP-seq data for CYS-3. Download Data Set S2, XLSX file, 0.04 MB. Copyright © 2021 Huberman et al. 2021 Huberman et al. https://creativecommons.org/licenses/by/4.0/ This content is distributed under the terms of the Creative Commons Attribution 4.0 International license.

**FIG 4:**
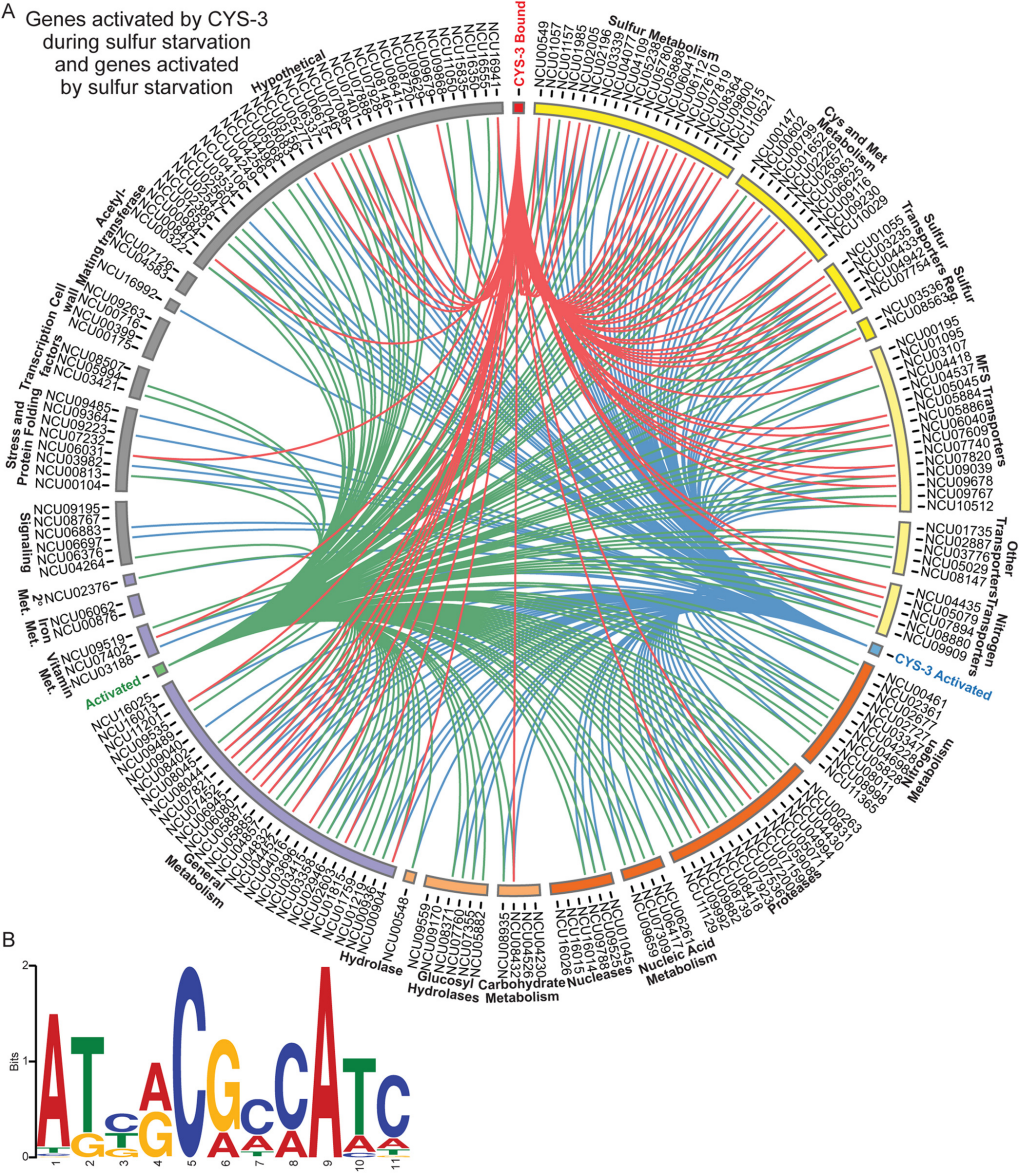
CYS-3 directly regulates genes involved in sulfur acquisition and metabolism. (A) Plot built with Circos version 0.69 (51) to display genes activated in response to sulfur starvation and/or by CYS-3. Green lines indicate genes that were expressed at least 2-fold higher in wild-type cells exposed to sulfur starvation than 24 μM sulfate. Blue lines indicate genes that were expressed at least 2-fold higher in wild-type than *cys-3^−^* cells exposed to sulfur starvation. Red lines indicate genes whose promoters were bound and whose expression was activated by CYS-3 in response to sulfur starvation. In this plot, all 62 genes whose promoters were bound by CYS-3 were activated by CYS-3, and 56 genes whose promoters were bound by CYS-3 were also activated in response to sulfur starvation. Of the 136 genes that were activated in response to sulfur starvation, 114 were activated by CYS-3. (B) CYS-3 consensus DNA binding motif (E value = 2.9 × 10^−39^) built using MEME version 5.1.1 (48).

Of the 643 genes with *in vitro* CYS-3 promoter binding sites, 64 were also differentially expressed by at least 2-fold between wild type and the *cys-3^−^* mutant. Functional enrichment analysis of these 64 genes showed overrepresentation of genes involved in sulfur, cysteine, methionine, glycine, serine, threonine, and selenocompound metabolism (Fig. 3A and C and Fig. 4A; Fig. S5 and Data Set S2). Expression of only two of these 64 genes increased in *cys-3^−^* cells compared to wild-type cells: *gly-3* (NCU02727) and glycosylphosphatidylinositol-anchored cell wall protein gene *tpd-1* (NCU00175) (38). The other 62 genes directly regulated by CYS-3 during sulfur starvation were activated by CYS-3, consistent with CYS-3 being a transcriptional activator (Fig. 4A; see Fig. S6 in the supplemental material).

10.1128/mSphere.00564-21.7 FIG S6 CYS-3 is primarily a transcriptional activator in response to sulfur starvation. Shown is a plot built with Circos, version 0.69 (51), to display genes repressed in response to sulfur starvation and/or by CYS-3. Green lines indicate genes that were expressed at least 2-fold lower in wild-type cells exposed to sulfur starvation than 24 μM sulfate. Blue lines indicate genes that were expressed at least 2-fold lower in wild-type than *cys-3^−^* cells exposed to sulfur starvation. Red lines indicate genes whose promoters were bound and whose expression was repressed at least 2-fold by CYS-3 in response to sulfur starvation. Download FIG S6, EPS file, 1.8 MB. Copyright © 2021 Huberman et al. 2021 Huberman et al. https://creativecommons.org/licenses/by/4.0/ This content is distributed under the terms of the Creative Commons Attribution 4.0 International license.

Using the 67 promoter binding sites in these 64 genes, we identified two potential consensus binding motifs: ATBRCGCCATC (E value = 2.9 × 10^−39^) and TTCYTYTYTYTTTKT (E value = 6.4 × 10^−3^) (Fig. 4B). The ATBRCGCCATC motif had a much smaller E value and significant similarities to the CYS-3 binding motif ATGGCGCCAT and ATGRYRYCAT, identified using random oligonucleotides (39) and DNA footprinting and mobility shift assays (40), respectively.

Promoters of genes involved in metabolizing nonpreferred sulfur sources were bound by CYS-3, and expression of these genes was regulated by CYS-3. These included arylsulfatase gene *ars-1*, choline sulfatase gene *chol-16*, cysteic acid decarboxylase gene *gdc-3*, dibenzothiophene desulfurization enzyme gene NCU05888, and taurine dioxygenase genes *tdo-2*, *tdo-3*, *tdo-4*, *tdo-5*, and *suc-2* (Fig. 2 and 4A). Of the two genes encoding alkanesulfonate monooxygenases, only *mox-7* was regulated by CYS-3. However, the promoters of *mox-7* and *aso-1* were both bound by CYS-3 in our unfiltered DAP-seq data. Although *aso-1* was not regulated in our RNA-seq data, the unfiltered DAP-seq data hint there may be a condition under which *aso-1* is expressed (Fig. 2; Data Set S2). Two genes involved in metabolizing sulfate were regulated by CYS-3, and the promoters of these two genes were bound by CYS-3: the gene coding for ATP sulfurylase, *cys-11*, which converts sulfate to APS, and the gene coding for PAPS reductase, *cys-5*, which converts PAPS to sulfite (Fig. 2). Additionally, CYS-3 both bound promoters and regulated expression of two genes involved in glutathione metabolism: *gst-4* and *gst-6* (Fig. 4A; Data Set S1).

Of genes directly involved in cysteine and methionine metabolism, only cystathionine γ-lyase gene *cys-16* and SAM synthetase gene *eth-1* were regulated by CYS-3 during sulfur starvation; promoters of both genes were bound by CYS-3 (Fig. 2 and 4A). The promoters of several genes involved in sulfur, cysteine, and methionine metabolism were bound by CYS-3 in our unfiltered DAP-seq data but were not regulated by CYS-3 under the conditions we tested. These included cystathionine β-synthase gene *met-11* (NCU09230), myo-inositol-1-monophosphatase gene *inl-3* (NCU09567), *utr4* (NCU06228), and methylenetetrahydrofolate reductase gene *met-13* (NCU09545) (Fig. 2; Data Set S2). Although we did not detect CYS-3-mediated regulation of these genes, the DAP-seq data hint CYS-3 may play a role in regulating sulfur metabolism at other times or conditions.

CYS-3 also bound promoters of all known and predicted sulfur transporter genes regulated by CYS-3. These included two sulfate permease genes (*cys-13* and *cys-14*), as well as chromate transporter gene *trm-50*. CYS-3 both regulated and bound the promoters of two predicted methionine permease genes, *aap-7* and *aap-10*. The promoter of a third predicted methionine permease gene, *aap-23*, was bound by CYS-3 in our unfiltered DAP-seq data, but *aap-23* expression was not regulated by CYS-3 or in response to sulfur starvation. This piece of DAP-seq binding data hints *aap-23* may play a role in sulfur metabolism under other conditions (Fig. 2 and 4A; Data Set S2).

Along with known and predicted sulfur transporters, CYS-3 both regulated and bound the promoters of other transporters in response to sulfur starvation. These included 8 MFS transporter genes (NCU07609, NCU09039, NCU05886, *mfs-15* [NCU05884], NCU09678, NCU10512, *mdr-7* [NCU01095], and predicted pantothenate transporter gene *mfs-29* [NCU07820]) (Fig. 4A). The direct activation of transporter gene expression by CYS-3 suggested one role of CYS-3 is regulating sulfur transport.

CYS-3 bound the promoters and regulated expression of three predicted amino acid or peptide transporter genes (*aap-18*, *aap-22*, and *mfs-9*). Additionally, CYS-3 directly regulated two protease genes: *mpr-4* (NCU05908) and *apr-10* (NCU08739) (Fig. 4A). Activation of these genes by CYS-3 may improve the ability of the cell to harvest sulfur from cysteine and methionine. However, promoters of most genes regulated by CYS-3 involved in nitrogen and carbon metabolism were not bound by CYS-3 in our DAP-seq data. This observation may suggest activation of these pathways is due to an indirect effect associated with nutrient stress.

Although promoters of genes in metabolic pathways associated with utilization of nutrient sources besides sulfur were not bound by CYS-3 in our DAP-seq data, the promoters of genes involved in oxidation-reduction reactions and other aspects of electron transfer were bound by CYS-3, and CYS-3 regulated their expression (Fig. 4A; Data Set S1). Iron-sulfur proteins play a role in oxidation-reduction reactions in electron transport in mitochondria. Direct regulation of genes involved in these general metabolic processes by CYS-3 suggests a connection between the sulfur response and ATP generation.

## DISCUSSION

Sulfur is a component of many essential biological macromolecules. To ensure a sufficient sulfur supply, filamentous fungi such as N. crassa scavenge sulfur from a number of sources, including cysteine, methionine, and other organic and inorganic sulfur compounds. Sulfur acquisition in N. crassa is regulated by the basic leucine zipper transcription factor CYS-3 (5). We used RNA-seq and DAP-seq to examine the global sulfur starvation response and the role of CYS-3 in regulating sulfur acquisition.

Our data indicate CYS-3 directly regulates most genes necessary for sulfur import and assimilation of sulfur from nonpreferred sulfur sources. Regulation of sulfur import and assimilation genes is similar to the role of CYS-3 homologs in other filamentous fungi. Transcriptional profiling of an A. fumigatus *metR* mutant under low sulfur showed MetR regulated expression of sulfate permeases, arylsulfatase, and genes involved in assimilating sulfur from sulfate (3). In A. nidulans, transcriptional activation of sulfur-responsive genes may be shared by MetR and its paralog, MetZ (12, 41).

### Sulfur import is an important aspect of the sulfur starvation response.

Sulfur sensing and utilization in N. crassa are well characterized using classical and molecular genetics. The transcription factor CYS-3 is responsible for activating the sulfur response (6). The *cys-3* mutant from the *Neurospora* deletion collection (33) has an identical phenotype to the previously characterized *cys-3* mutant and is required for regulation of genes previously identified to require CYS-3 (Fig. S1 and S4 and Data Set S1) (6, 34). Importantly, our DAP-seq data show CYS-3 binds promoters of genes expressed upon sulfur starvation (Fig. 4A). However, as both characterized *cys-3* mutants are not the result of complete deletions (Fig. S4) (6), we cannot rule out the possibility that the truncated CYS-3 proteins may play an additional role in the cell.

DAP-seq is comparable to the *in vivo* genome-wide method of identifying DNA binding sites, chromatin immunoprecipitation sequencing, in N. crassa and *Arabidopsis*, with a high concordance of identified transcription factor DNA binding sites (18, 37). We identified 64 genes whose promoters were bound by CYS-3 in our DAP-seq data and were at least 2-fold differentially expressed when comparing wild-type and *cys-3^−^* cells exposed to sulfur starvation (Fig. 4A; Fig. S6). This set of genes included a number of genes known or predicted to play a role in sulfur metabolism, as well as uncharacterized transporters and genes involved in electron transport. However, this list likely does not include all genes CYS-3 directly regulates. We identified 6 genes (*aso-1*, *met-11*, *inl-3*, *utr4*, *met-13*, and *aap-23*) known or predicted to play roles in sulfur metabolism whose promoters were bound by CYS-3 in our unfiltered DAP-seq data set but were not differentially expressed in our 4-h RNA-seq data set (Data Set S1 and S2). It is likely additional genes that are bound by CYS-3 and require CYS-3 for regulation would be identified under different nutrient conditions and time points than those examined here. Our DAP-seq data provide information that will guide these future experimental approaches. The combination of transcriptional profiling under different nutrient conditions, other mutants, and improved DAP-seq library methods may identify additional genes that are directly regulated by CYS-3.

A small number of previously identified CYS-3 DNA binding sites were not identified using DAP-seq. Several studies using mobility shift assays and DNA footprinting show CYS-3 binds its own promoter (5, 35, 36, 40, 42). However, CYS-3 binding sites were not identified in the *cys-3* promoter using DAP-seq (Data Set S2). CYS-3 protein levels are regulated by SCON-2 when sulfur is abundant (7, 8). *scon-2* is regulated by CYS-3 (Fig. S3) (43), and prior studies identified 4 CYS-3 binding sites in the *scon-2* promoter (43). We identified multiple CYS-3 binding sites in the *scon-2* promoter, but they did not overlap the 4 sites identified by mobility shift assays (Data Set S2).

Differences in DAP-seq and mobility shift or DNA footprinting assay transcription factor binding site identification may be due to differing methods of protein and DNA preparation. We generated CYS-3 protein for DAP-seq using *in vitro* transcription translation. Previous studies expressed CYS-3 in Escherichia coli or harvested CYS-3 from N. crassa nuclear extracts (5, 35, 36, 40, 42). Protein modifications of CYS-3 that occur *in vivo* may affect DNA binding. Additionally, earlier studies used a mixture of genomic DNA, synthesized oligonucleotides, and cloned DNA fragments (5, 35, 36, 40, 42); we used genomic DNA from N. crassa grown in VMM. It is possible that DNA modifications may affect CYS-3 DNA binding. For example, DNA methylation can block CYS-3 binding of the *cys-14* promoter, although the same effect was not seen for the *cys-3* promoter (40).

Our transcriptional data provide a global view of the sulfur response in N. crassa. During sulfur starvation, CYS-3 directly upregulates many genes required for sulfur import. We identified CYS-3 binding sites in promoters of known and predicted sulfur transporters, such as sulfate permease genes *cys-13* and *cys-14* and methionine permease genes *aap-7* and *aap-10*, as well as predicted amino acid and peptide transporter genes and 8 uncharacterized MFS transporter genes (Fig. 2 and 4A; Data Set S2). We confirmed CYS-3 binding 1.4 kbp upstream of the *cys-14* translation start site (35) (Data Set S2), which is necessary and sufficient for full, regulated expression of *cys-14* (44). The direct regulation of so many transporters by CYS-3 suggests sulfur import is an important aspect of the sulfur starvation response.

### Cross talk exists between regulation of sulfur metabolism and a variety of other primary metabolic pathways.

In nature, N. crassa gets most of its nutrients from plant biomass, where sulfur is in a matrix of other nutrients. Our data indicate the sulfur response is transcriptionally intertwined with other metabolic pathways. Along with direct regulation of genes involved in sulfur, cysteine, and methionine metabolism, CYS-3 also directly regulates genes involved in electron transfer, perhaps due to the presence of sulfur in a number of electron carriers, such as iron-sulfur proteins (Fig. 4A). The requirement for iron-sulfur proteins for cell viability may also be the reason genes involved in iron acquisition are regulated by the A. fumigatus CYS-3 ortholog MetR (3). However, in N. crassa, indirect regulation of only a small number of genes involved in iron metabolism was identified (Fig. 4A; Fig. S6 and Data Set S1). Direct regulation of many genes involved in oxidation-reduction reactions and electron transport by CYS-3 may suggest a connection between the sulfur response and metabolic pathways regulating ATP generation.

Additionally, genes involved in carbon and nitrogen metabolism were regulated during sulfur starvation (Fig. 4A; Fig. S6). Coregulation of nutrient metabolism is not restricted to N. crassa, as similar phenomena are observed in the human pathogens A. fumigatus and Cryptococcus neoformans (3, 45). Our DAP-seq data suggest the interplay between these signaling pathways may not be directly regulated by CYS-3. It will be the role of future studies to investigate the molecular mechanisms controlling cross talk between these nutrient sensing and metabolism pathways.

## MATERIALS AND METHODS

### N. crassa strains and culturing.

The strains used in this study were wild-type *mat* A and *mat* a strains FGSC 2489 and FGSC 4200, respectively (wild type) (46) and FGSC 26799 *cys-3^−^*::*hyg*^R^ *mat* a mutant strain (*cys-3^−^*) (this study). For details of FGSC 26799 construction, see Text S1 in the supplemental material.

10.1128/mSphere.00564-21.1 TEXT S1 Supplemental materials and methods. Download Text S1, DOCX file, 0.03 MB. Copyright © 2021 Huberman et al. 2021 Huberman et al. https://creativecommons.org/licenses/by/4.0/ This content is distributed under the terms of the Creative Commons Attribution 4.0 International license.

The media used in this study were based on VMM, which contains 58 mM sucrose as the carbon source, 25 mM ammonium nitrate as the nitrogen source, and 800 μM sulfate as the sulfur source (19). For details of the recipes of the media used and N. crassa growth conditions, see Text S1. All chemicals were purchased from Sigma-Aldrich unless otherwise noted.

### RNA sequencing and transcript abundance.

The methods of RNA extraction, library preparation, and sequencing were modified from those of Wu et al. (18). For details, see Text S1.

### Statistical significance tests.

RNA-seq experiments had 3 biological replicates, and statistical significance was determined using Cufflinks v2.2.1 (47). Biological replicates refer to independent cultures inoculated on the same or independent days.

### DAP-seq.

Single DAP-seq libraries were prepared for CYS-3 and a negative control as described by Wu et al. (18). DAP-seq data were filtered for CYS-3 DNA binding sites within 3,000 bp upstream of a translational start site. This list was further filtered to only include genes whose expression was at least 2-fold differentially regulated between wild-type and *cys-3^−^* cells during exposure to sulfur starvation or 24 μM sulfate with a count of fragments per kilobase per million (FPKM) of at least 10 under any one of these conditions. For details, see Text S1.

### DNA binding consensus motif generation.

Motif discovery was performed using MEME version 5.1.1 (48). For details, see Text S1.

### Functional enrichment analysis and gene annotation.

Functional enrichment analysis was done using FungiFun2 (https://elbe.hki-jena.de/fungifun/) with KEGG as the classification ontology (49, 50). Gene to category associates were tested for overrepresentation using hypergeometric distribution with Benjamini-Hochberg correction for false-discovery rate.

Gene annotations were pulled from FungiDB (https://fungidb.org/) or inferred from homology to characterized genes in related fungi.

### Data availability.

The RNA-seq data used in this study have been deposited in the Gene Expression Omnibus (GEO) at the National Center for Biotechnology Information (NCBI) and are accessible through GEO series accession no. GSE173890. Processed RNA-seq data are available in Data Set S1. DAP-seq data used in this study have been deposited in the NCBI Sequence Read Archive (SRA) and are accessible through SRA accession no. SRX3748478 (CYS-3) and SRX3748477 (negative control). Processed DAP-seq data are available in Data Set S2.

10.1128/mSphere.00564-21.8 TABLE S1 Sulfur-free trace elements solution (100 ml). One hundred microliters of sulfur-free trace elements solution was added to each liter of sulfur-free VMM. Download Table S1, DOCX file, 0.02 MB. Copyright © 2021 Huberman et al. 2021 Huberman et al. https://creativecommons.org/licenses/by/4.0/ This content is distributed under the terms of the Creative Commons Attribution 4.0 International license.

## References

[1] Hästbacka J, de la Chapelle A, Mahtani MM, Clines G, Reeve-Daly MP, Daly M, Hamilton BA, Kusumi K, Trivedi B, Weaver A. 1994. The diastrophic dysplasia gene encodes a novel sulfate transporter: positional cloning by fine-structure linkage disequilibrium mapping. Cell 78:1073–1087. doi:10.1016/0092-8674(94)90281-x. 7923357

[2] Franco B, Meroni G, Parenti G, Levilliers J, Bernard L, Gebbia M, Cox L, Maroteaux P, Sheffield L, Rappold GA, Andria G, Petit C, Ballabio A. 1995. A cluster of sulfatase genes on Xp22.3: mutations in chondrodysplasia punctata (CDPX) and implications for warfarin embryopathy. Cell 81:15–25. doi:10.1016/0092-8674(95)90367-4. 7720070

[3] Amich J, Schafferer L, Haas H, Krappmann S. 2013. Regulation of sulphur assimilation is essential for virulence and affects iron homeostasis of the human-pathogenic mould *Aspergillus fumigatus*. PLoS Pathog 9:e1003573. doi:10.1371/journal.ppat.1003573. 24009505 PMC3757043

[4] Marzluf GA. 1997. Molecular genetics of sulfur assimilation in filamentous fungi and yeast. Annu Rev Microbiol 51:73–96. doi:10.1146/annurev.micro.51.1.73. 9343344

[5] Paietta JV, Akins RA, Lambowitz AM, Marzluf GA. 1987. Molecular cloning and characterization of the *cys-3* regulatory gene of *Neurospora crassa*. Mol Cell Biol 7:2506–2511. doi:10.1128/mcb.7.7.2506-2511.1987. 2886908 PMC365384

[6] Paietta JV. 1992. Production of the CYS3 regulator, a bZIP DNA-binding protein, is sufficient to induce sulfur gene expression in *Neurospora crassa*. Mol Cell Biol 12:1568–1577. doi:10.1128/mcb.12.4.1568-1577.1992. 1532230 PMC369599

[7] Kumar A, Paietta JV. 1998. An additional role for the F-box motif: gene regulation within the *Neurospora crassa* sulfur control network. Proc Natl Acad Sci USA 95:2417–2422. doi:10.1073/pnas.95.5.2417. 9482900 PMC19360

[8] Sizemore ST, Paietta JV. 2002. Cloning and characterization of *scon-3*+, a new member of the *Neurospora crassa* sulfur regulatory system. Eukaryot Cell 1:875–883. doi:10.1128/EC.1.6.875-883.2002. 12477788 PMC138751

[9] Natorff R, Sieńko M, Brzywczy J, Paszewski A. 2003. The *Aspergillus nidulans metR* gene encodes a bZIP protein which activates transcription of sulphur metabolism genes. Mol Microbiol 49:1081–1094. doi:10.1046/j.1365-2958.2003.03617.x. 12890030

[10] Natorff R, Piotrowska M, Paszewski A. 1998. The Aspergillus nidulans sulphur regulatory gene sconB encodes a protein with WD40 repeats and an F-box. Mol Gen Genet 257:255–263. doi:10.1007/s004380050646. 9520259

[11] Piotrowska M, Natorff R, Paszewski A. 2000. *sconC*, a gene involved in the regulation of sulphur metabolism in *Aspergillus nidulans*, belongs to the SKP1 gene family. Mol Gen Genet 264:276–282. doi:10.1007/s004380000319. 11085267

[12] Piłsyk S, Natorff R, Sieńko M, Skoneczny M, Paszewski A, Brzywczy J. 2015. The *Aspergillus nidulans metZ* gene encodes a transcription factor involved in regulation of sulfur metabolism in this fungus and other *Eurotiales*. Curr Genet 61:115–125. doi:10.1007/s00294-014-0459-5. 25391366

[13] Ketter JS, Jarai G, Fu YH, Marzluf GA. 1991. Nucleotide sequence, messenger RNA stability, and DNA recognition elements of *cys-14*, the structural gene for sulfate permease II in *Neurospora crassa*. Biochemistry 30:1780–1787. doi:10.1021/bi00221a008. 1825178

[14] Hanson MA, Marzluf GA. 1975. Control of the synthesis of a single enzyme by multiple regulatory circuits in *Neurospora crassa*. Proc Natl Acad Sci USA 72:1240–1244. doi:10.1073/pnas.72.4.1240. 124058 PMC432507

[15] Marzluf GA. 1972. Genetic and metabolic control of sulfate metabolism in *Neurospora crassa*: a specific permease for choline-*O*-sulfate. Biochem Genet 7:219–233. doi:10.1007/BF00484820. 4265021

[16] Paietta JV. 1989. Molecular cloning and regulatory analysis of the arylsulfatase structural gene of *Neurospora crassa*. Mol Cell Biol 9:3630–3637. doi:10.1128/mcb.9.9.3630-3637.1989. 2528685 PMC362423

[17] Huberman LB, Wu VW, Kowbel DJ, Lee J, Daum C, Grigoriev IV, O'Malley RC, Glass NL. 2021. DNA affinity purification sequencing and transcriptional profiling reveal new aspects of nitrogen regulation in a filamentous fungus. Proc Natl Acad Sci USA 118:e2009501118. doi:10.1073/pnas.2009501118. 33753477 PMC8020665

[18] Wu VW, Thieme N, Huberman LB, Dietschmann A, Kowbel DJ, Lee J, Calhoun S, Singan VR, Lipzen A, Xiong Y, Monti R, Blow MJ, O'Malley RC, Grigoriev IV, Benz JP, Glass NL. 2020. The regulatory and transcriptional landscape associated with carbon utilization in a filamentous fungus. Proc Natl Acad Sci USA 117:6003–6013. doi:10.1073/pnas.1915611117. 32111691 PMC7084071

[19] Vogel H. 1956. A convenient growth medium for *Neurospora* (medium N). Microbial Genet Bull 13:42–43.

[20] Marzluf GA. 1994. Genetics and molecular genetics of sulfur assimilation in the fungi. Adv Genet 31:187–206. doi:10.1016/s0065-2660(08)60398-3. 8036994

[21] Flavin M, Slaughter C. 1967. The derepression and function of enzymes of reverse trans-sulfuration in *Neurospora*. Biochim Biophys Acta 132:406–411. doi:10.1016/0005-2744(67)90159-3. 6031132

[22] Marzluf GA, Metzenberg RL. 1968. Positive control by the *cys-3* locus in regulation of sulfur metabolism in *Neurospora*. J Mol Biol 33:423–437. doi:10.1016/0022-2836(68)90199-x. 5700703

[23] Metzenberg RL, Parson JW. 1966. Altered repression of some enzymes of sulfur utilization in a temperature-conditional lethal mutant of *Neurospora*. Proc Natl Acad Sci USA 55:629–635. doi:10.1073/pnas.55.3.629. 5221246 PMC224198

[24] Paietta JV. 2016. Regulation of sulfur metabolism in filamentous fungi, p 305–319. *In* Hoffmeister D (ed), The Mycota: biochemistry and molecular biology, 3rd ed. Springer, Berlin, Germany.

[25] Marzluf GA. 1970. Genetic and biochemical studies of distinct sulfate permease species in different developmental stages of *Neurospora crassa*. Arch Biochem Biophys 138:254–263. doi:10.1016/0003-9861(70)90306-1. 5446339

[26] Marzluf GA. 1970. Genetic and metabolic controls for sulfate metabolism in *Neurospora crassa*: isolation and study of chromate-resistant and sulfate transport-negative mutants. J Bacteriol 102:716–721. doi:10.1128/jb.102.3.716-721.1970. 5429722 PMC247617

[27] Flores-Alvarez LJ, Corrales-Escobosa AR, Cortés-Penagos C, Martínez-Pacheco M, Wrobel-Zasada K, Wrobel-Kaczmarczyk K, Cervantes C, Gutiérrez-Corona F. 2012. The *Neurospora crassa chr-1* gene is up-regulated by chromate and its encoded CHR-1 protein causes chromate sensitivity and chromium accumulation. Curr Genet 58:281–290. doi:10.1007/s00294-012-0383-5. 23085746

[28] Apodaca G, McKerrow JH. 1989. Regulation of *Trichophyton rubrum* proteolytic activity. Infect Immun 57:3081–3090. doi:10.1128/iai.57.10.3081-3090.1989. 2476398 PMC260773

[29] Cohen BL. 1973. Regulation of intracellular and extracellular neutral and alkaline proteases in *Aspergillus nidulans*. J Gen Microbiol 79:311–320. doi:10.1099/00221287-79-2-311. 4589332

[30] Katz ME, Rice RN, Cheetham BF. 1994. Isolation and characterization of an *Aspergillus nidulans* gene encoding an alkaline protease. Gene 150:287–292. doi:10.1016/0378-1119(94)90439-1. 7821793

[31] Benz JP, Chau BH, Zheng D, Bauer S, Glass NL, Somerville CR. 2014. A comparative systems analysis of polysaccharide-elicited responses in *Neurospora crassa* reveals carbon source-specific cellular adaptations. Mol Microbiol 91:275–299. doi:10.1111/mmi.12459. 24224966 PMC3900418

[32] Paietta JV. 1990. Molecular cloning and analysis of the *scon-2* negative regulatory gene of *Neurospora crassa*. Mol Cell Biol 10:5207–5214. doi:10.1128/mcb.10.10.5207-5214.1990. 1975945 PMC361201

[33] Colot HV, Park G, Turner GE, Ringelberg C, Crew CM, Litvinkova L, Weiss RL, Borkovich KA, Dunlap JC. 2006. A high-throughput gene knockout procedure for *Neurospora* reveals functions for multiple transcription factors. Proc Natl Acad Sci USA 103:10352–10357. doi:10.1073/pnas.0601456103. 16801547 PMC1482798

[34] Reveal BS, Paietta JV. 2012. Analysis of the sulfur-regulated control of the cystathionine γ-lyase gene of *Neurospora crassa*. BMC Res Notes 5:339. doi:10.1186/1756-0500-5-339. 22748183 PMC3496659

[35] Fu YH, Marzluf GA. 1990. *cys-3*, the positive-acting sulfur regulatory gene of *Neurospora crassa*, encodes a sequence-specific DNA-binding protein. J Biol Chem 265:11942–11947. doi:10.1016/S0021-9258(19)38491-1. 2142156

[36] Kanaan MN, Marzluf GA. 1993. The positive-acting sulfur regulatory protein CYS3 of *Neurospora crassa*: nuclear localization, autogenous control, and regions required for transcriptional activation. Mol Gen Genet 239:334–344. doi:10.1007/BF00276931. 8316209

[37] O'Malley RC, Huang SC, Song L, Lewsey MG, Bartlett A, Nery JR, Galli M, Gallavotti A, Ecker JR. 2016. Cistrome and epicistrome features shape the regulatory DNA landscape. Cell 165:1280–1292. doi:10.1016/j.cell.2016.04.038. 27203113 PMC4907330

[38] Maddi A, Free SJ. 2010. α-1,6-Mannosylation of N-linked oligosaccharide present on cell wall proteins is required for their incorporation into the cell wall in the filamentous fungus *Neurospora crassa*. Eukaryot Cell 9:1766–1775. doi:10.1128/EC.00134-10. 20870880 PMC2976294

[39] Paietta JV. 2008. DNA-binding specificity of the CYS3 transcription factor of *Neurospora crassa* defined by binding-site selection. Fungal Genet Biol 45:1166–1171. doi:10.1016/j.fgb.2008.05.001. 18565773

[40] Li Q, Marzluf GA. 1996. Determination of the *Neurospora crassa* CYS 3 sulfur regulatory protein consensus DNA-binding site: amino-acid substitutions in the CYS3 bZIP domain that alter DNA-binding specificity. Curr Genet 30:298–304. doi:10.1007/s002940050136. 8781172

[41] Sieńko M, Natorff R, Skoneczny M, Kruszewska J, Paszewski A, Brzywczy J. 2014. Regulatory mutations affecting sulfur metabolism induce environmental stress response in *Aspergillus nidulans*. Fungal Genet Biol 65:37–47. doi:10.1016/j.fgb.2014.02.001. 24513272

[42] Kanaan MN, Marzluf GA. 1991. Mutational analysis of the DNA-binding domain of the CYS3 regulatory protein of *Neurospora crassa*. Mol Cell Biol 11:4356–4362. doi:10.1128/mcb.11.9.4356-4362.1991. 1831537 PMC361297

[43] Kumar A, Paietta JV. 1995. The sulfur controller-2 negative regulatory gene of *Neurospora crassa* encodes a protein with beta-transducin repeats. Proc Natl Acad Sci USA 92:3343–3347. doi:10.1073/pnas.92.8.3343. 7724564 PMC42162

[44] Li Q, Zhou L, Marzluf GA. 1996. Functional in vivo studies of the *Neurospora crassa cys-14* gene upstream region: importance of CYS3-binding sites for regulated expression. Mol Microbiol 22:109–117. doi:10.1111/j.1365-2958.1996.tb02660.x. 8899713

[45] de Melo AT, Martho KF, Roberto TN, Nishiduka ES, Machado J, Jr, Brustolini OJB, Tashima AK, Vasconcelos AT, Vallim MA, Pascon RC. 2019. The regulation of the sulfur amino acid biosynthetic pathway in *Cryptococcus neoformans*: the relationship of Cys3, calcineurin, and Gpp2 phosphatases. Sci Rep 9:11923. doi:10.1038/s41598-019-48433-5. 31417135 PMC6695392

[46] McCluskey K, Wiest A, Plamann M. 2010. The Fungal Genetics Stock Center: a repository for 50 years of fungal genetics research. J Biosci 35:119–126. doi:10.1007/s12038-010-0014-6. 20413916

[47] Trapnell C, Roberts A, Goff L, Pertea G, Kim D, Kelley DR, Pimentel H, Salzberg SL, Rinn JL, Pachter L. 2012. Differential gene and transcript expression analysis of RNA-seq experiments with TopHat and Cufflinks. Nat Protoc 7:562–578. doi:10.1038/nprot.2012.016. 22383036 PMC3334321

[48] Bailey TL, Boden M, Buske FA, Frith M, Grant CE, Clementi L, Ren J, Li WW, Noble WS. 2009. MEME SUITE: tools for motif discovery and searching. Nucleic Acids Res 37:W202–W208. doi:10.1093/nar/gkp335. 19458158 PMC2703892

[49] Priebe S, Kreisel C, Horn F, Guthke R, Linde J. 2015. FungiFun2: a comprehensive online resource for systematic analysis of gene lists from fungal species. Bioinformatics 31:445–446. doi:10.1093/bioinformatics/btu627. 25294921 PMC4308660

[50] Kanehisa M, Goto S. 2000. KEGG: Kyoto Encyclopedia of Genes and Genomes. Nucleic Acids Res 28:27–30. doi:10.1093/nar/28.1.27. 10592173 PMC102409

[51] Krzywinski M, Schein J, Birol I, Connors J, Gascoyne R, Horsman D, Jones SJ, Marra MA. 2009. Circos: an information aesthetic for comparative genomics. Genome Res 19:1639–1645. doi:10.1101/gr.092759.109. 19541911 PMC2752132

