# Supplementary figures and images for: Aspects of the Neurospora crassa Sulfur Starvation Response Are Revealed by Transcriptional Profiling and DNA Affinity Purification Sequencing
Source: mSphere. 2021 Sep 15;6(5):e00564-21. doi: 10.1128/mSphere.00564-21 (PMC8550094; doi:10.1128/mSphere.00564-21)

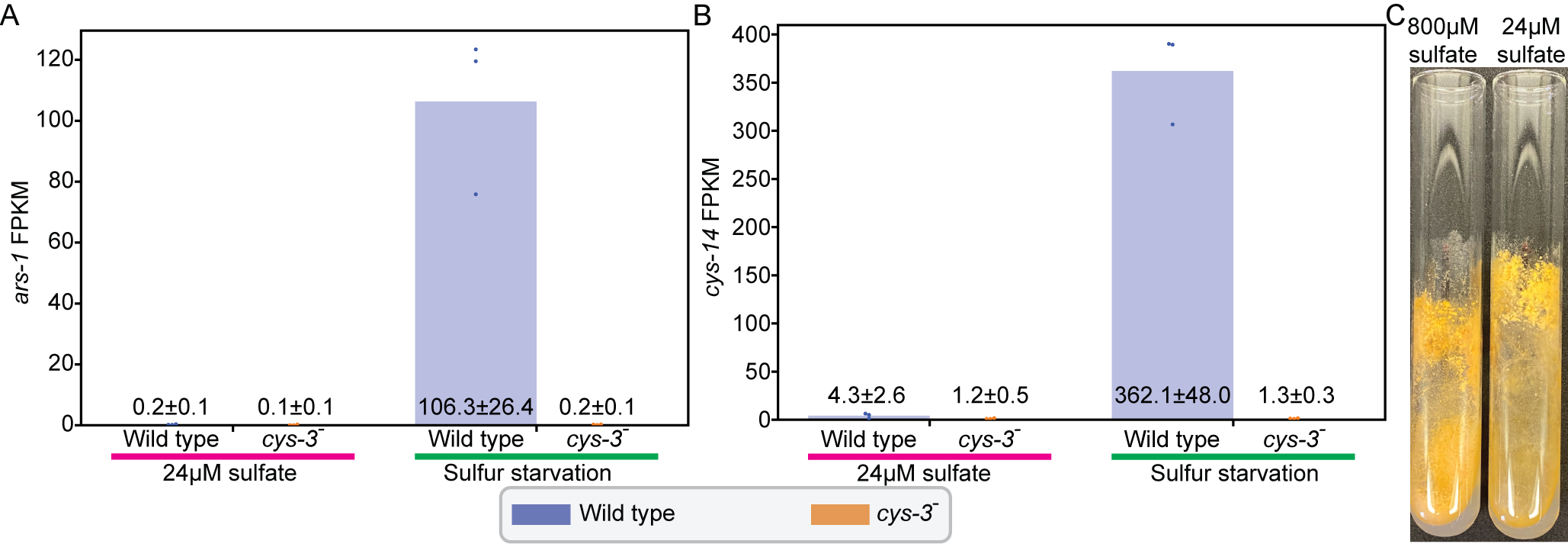

Supplement: FIG S1 [file msphere.00564-21-sf001.tif]

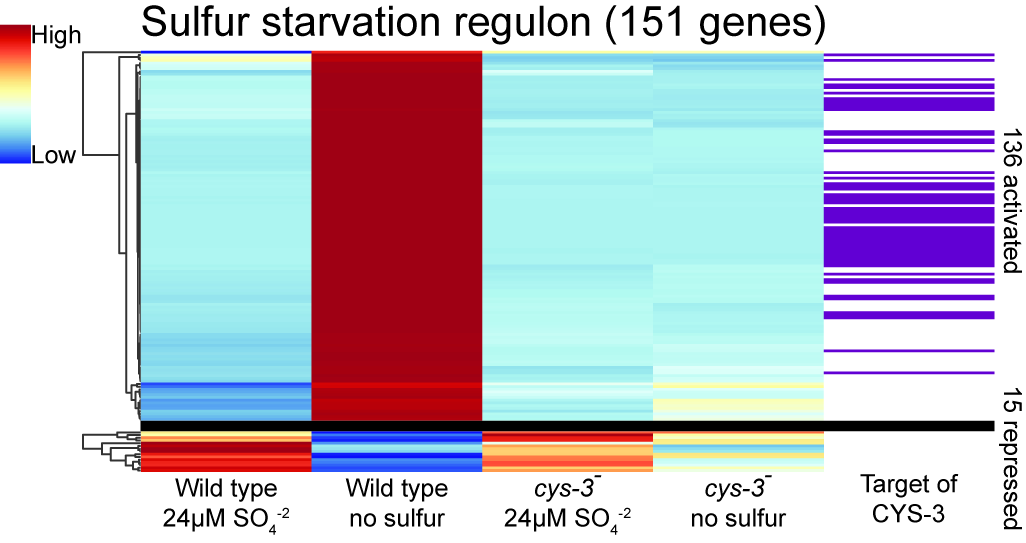

Supplement: FIG S2 [file msphere.00564-21-sf002.tif]

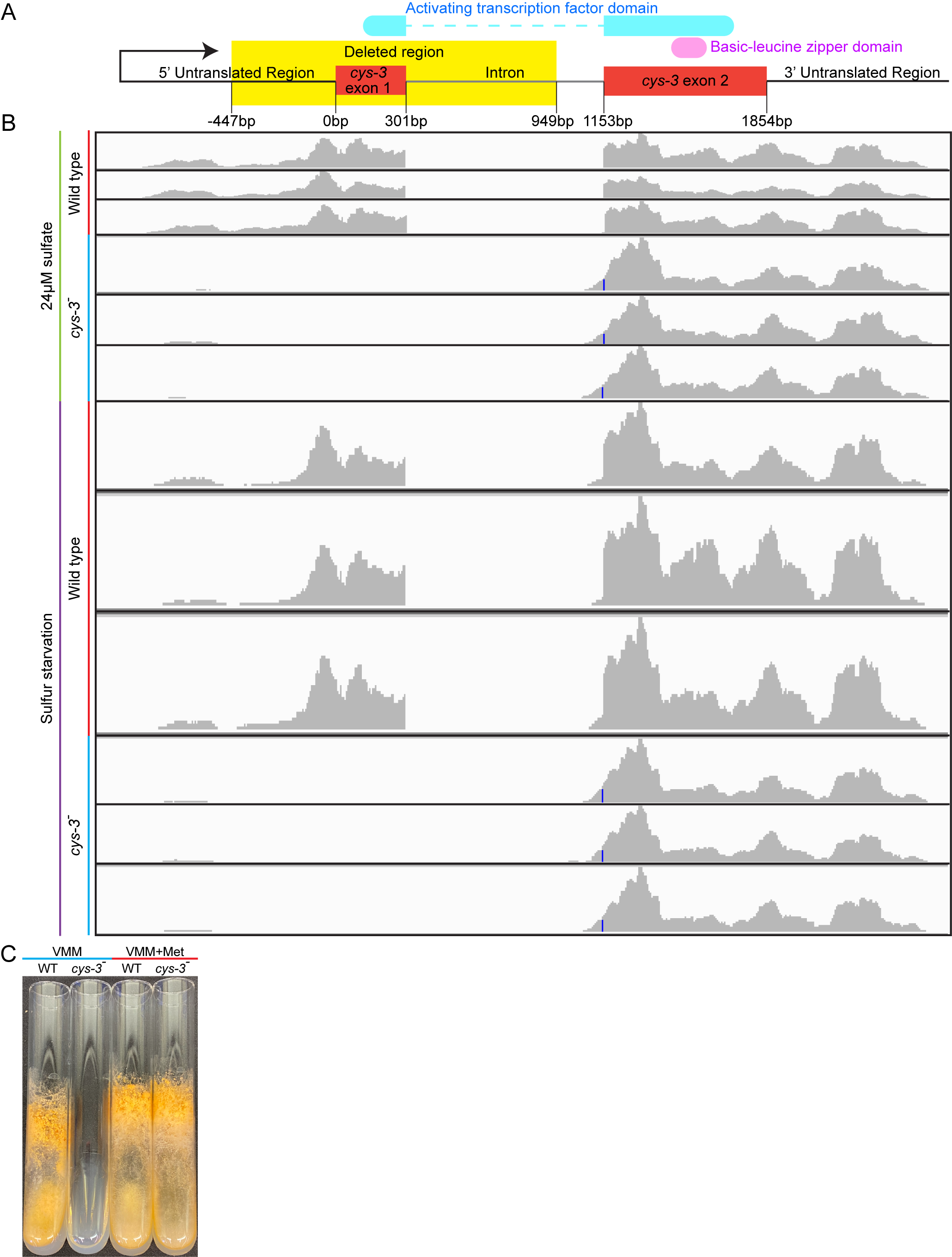

Supplement: FIG S4 [file msphere.00564-21-sf004.tif]

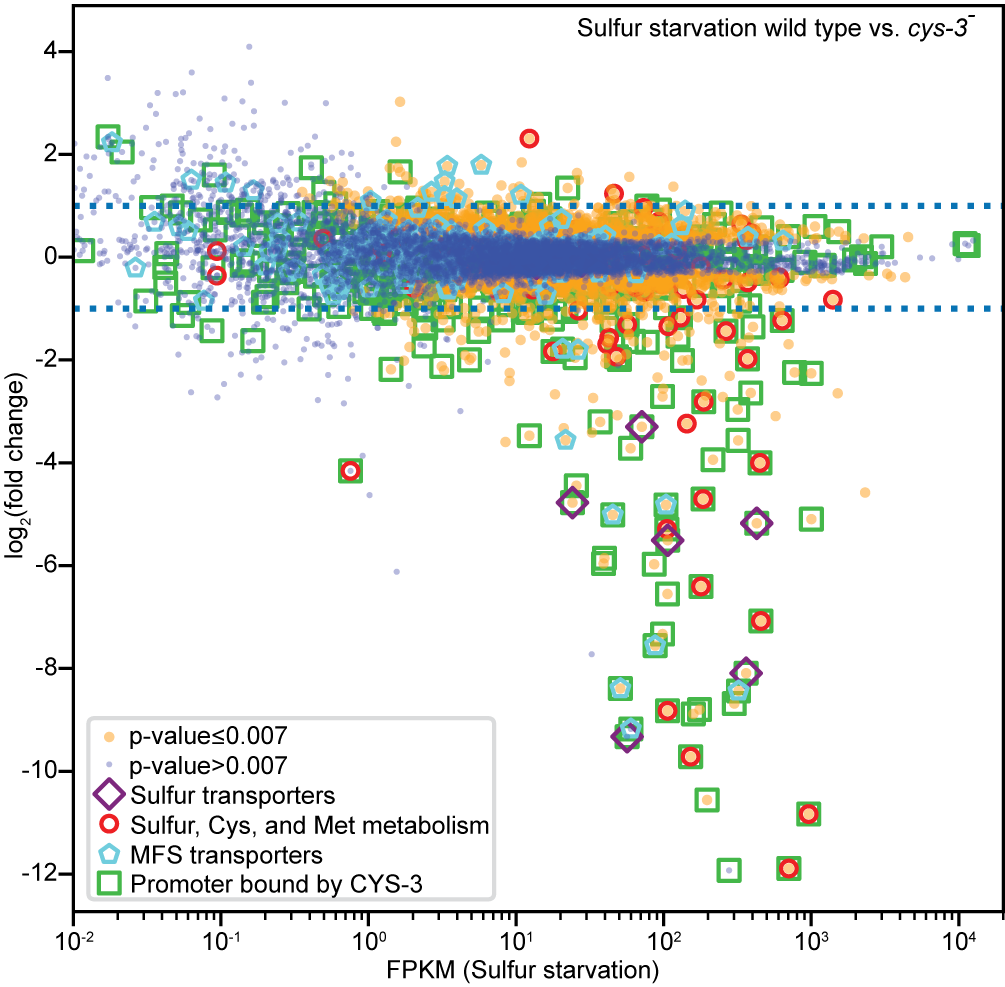

Supplement: FIG S5 [file msphere.00564-21-sf005.tif]
