# Supplementary material for: Aspects of the Neurospora crassa Sulfur Starvation Response Are Revealed by Transcriptional Profiling and DNA Affinity Purification Sequencing
Source: mSphere. 2021 Sep 15;6(5):e00564-21. doi: 10.1128/mSphere.00564-21 (PMC8550094; doi:10.1128/mSphere.00564-21)
Supplement: TEXT S1 [file msphere.00564-21-s0001.docx]

**SI MATERIALS and METHODS**

***N. crassa strains and culturing***

Strains used in this study were FGSC 2489, the wild type *mat* A strain (wild type) (1), FGSC 4200, the wild type *mat a* strain (wild type) (1), and FGSC 26799, the *cys-3*^-^*::hyg^R^ mat a* strain (*cys-3*^-^) (this study). The homokaryotic *cys-3*^-^ strain (FGSC 26799) was derived from the heterokaryotic FGSC 21610 from the *N. crassa* deletion collection by isolating uninucleate microconidia (1, 2). As described in the construction of the *N. crassa* deletion collection, in the *cys-3^-^* strain (FGSC 26799), the hygromycin resistance cassette replaced a portion of the 5’ untranslated region, the first *cys-3* exon (301 base pairs), and a portion of the *cys-3* intron (Fig. S4). The deleted region was bounded by the sequences 5’-GCTCCAGTCTCAATATCTCC-3’ (upstream of deletion) and 5’- TACCCGTCTTGGTTACTACG-3’ (downstream of deletion) (2). We confirmed the presence of the hygromycin resistance cassette at this locus using PCR with the primers 5’-CTTGTCTCGACCAGGAGTC-3’ upstream of *cys-3* and 5’-GACCGATGGCTGTGTAGAAGTACTC-3’ in the hygromycin cassette.

The media used in this study were based on the Vogel’s minimal medium (VMM) recipe, which contains 58mM sucrose as the carbon source, 25mM ammonium nitrate as the nitrogen source, and 800μM sulfate as the sulfur source (3). To make VMM containing 24μM sulfate, a sulfur concentration that supports growth of *N. crassa* and represses expression of sulfur responsive genes (Fig. S1), 165mg/L magnesium chloride hexahydrate replaced the magnesium sulfate heptahydrate. For sulfur starvation experiments, 165mg/L magnesium chloride hexahydrate replaced the magnesium sulfate heptahydrate and all of the sulfate salts in the trace elements solution were replaced with chloride salts (Table S1).

For RNAseq experiments, FGSC 4200 and FGSC 26799 were grown from freezer stocks on VMM + 500mg/L methionine + 1.5% agar (Thermo Fisher Scientific) slants for 2 d at 30^o^C in the dark and 8 d at 25^o^C in constant light. Conidia were harvested from slants and inoculated into 3ml VMM + 500mg/L methionine in 24-well plates at 10^6^ conidia/mL and grown for 24 h at 25^o^C in constant light with shaking at 200 rpm. The media was then vacuumed out of the wells and mycelial mats were washed three times with either VMM containing 24μM sulfate or VMM lacking a sulfur source and then shifted to 3 ml of either VMM containing 24μM sulfate or VMM lacking a sulfur source, respectively. Cells were then incubated for 4 h at 25^o^C in constant light with shaking at 200 rpm prior to harvesting by flash freezing in liquid nitrogen to harvest RNA for RNAseq.

For growth experiments, *N. crassa* strains were inoculated from freezer stocks on VMM containing the indicated sulfur conditions (800μM sulfate, 800μM sulfate + 500mg/L methionine, or 24μM sulfate) + 1.5% agar slants and grown for 2 d at 30^o^C in constant dark and 4 d at 25^o^C in constant light. All chemicals were purchased from Sigma-Aldrich unless otherwise noted.

***RNA sequencing and transcript abundance***

FGSC 4200 and FGSC 26799 conidia were inoculated into 3ml VMM + 500mg/L methionine in 24-well plates at 10^6^ conidia/mL and grown for 24 h at 25^o^C in constant light with shaking at 200 rpm. The media was then vacuumed out of the wells and mycelial mats were washed three times with either VMM containing 24μM sulfate or VMM lacking a sulfur source and then shifted to 3 ml of either VMM containing 24μM sulfate or VMM lacking a sulfur source, respectively. Cells were then incubated for 4 h at 25^o^C in constant light with shaking at 200 rpm.

Mycelia were harvested by filtering on Whatman paper no. 1 and flash frozen in liquid nitrogen. RNA extraction and library preparation were done as described in Wu *et al* (2020) (4). Briefly, total RNA was harvested from mycelia flash frozen in liquid nitrogen 4h post-transfer to the indicated media using a TRIzol (Life Technologies) extraction and cleaned up with the RNeasy kit (Qiagen). Libraries were prepared from total RNA using poly(A) enrichment and standard Illumina protocols. Libraries were prepared and sequenced at the UC Davis Genome Center on an Illumina HiSeq 4000 with 90bp single end reads.

The RNA sequences were aligned to the predicted transcripts from the *N. crassa* OR74A genome (v12) (5) with HiSat2 v2.0.5 (6) with the maximum intron length set to 5000bp. Transcript abundance (fragments per kilobase of transcript per million mapped reads, FPKM) and differential expression were determined using Cufflinks v2.2.1 (7) with the options --library-type fr-unstranded, --max-bundle-frags=10000000, and --compatible-hits-norm.

Hierarchical clustering was performed using the clustermap function of the Python visualization library Seaborn (<https://seaborn.pydata.org/>) using the average linkage method with Pearson Correlation as the similarity metric.

RNAseq data used in this study were deposited in the Gene Expression Omnibus (GEO) at the National Center for Biotechnology Information (NCBI) and are accessible through GEO series accession number GSE173890. Processed RNAseq data are available in Dataset S1.

***DAPseq***

DAPseq was done as described in Wu *et al* (2020) (4). Briefly, the *cys-3* open reading frame was amplified from cDNA using RNA to cDNA EcoDry premix (Clontech) and inserted into an expression vector containing T7 and SP6 promoters upstream of a HALO tag as previously described (8). The *cys-3* transcription factor was transcribed and translated *in vitro* using Promega TnT T7 Rabbit Reticulocyte Quick Coupled Transcription/Translation System and expression was verified by Western blot with the Promega Anti-HaloTag monoclonal antibody. The CYS-3 protein was then bound to Promega Magne HaloTag Beads.

Genomic DNA was harvested from wild type *N. crassa* mycelia grown in liquid VMM containing 800μM sulfate for 25h at 25^o^C using the DNeasy Blood & Tissue Kit (Qiagen). DNA was sheared to a 300bp peak and size selected using AMPure XP beads (Beckman Coulter). *In vitro* translated CYS-3 was bound to Promega Magne HaloTag Beads and incubated with genomic DNA on a rotator for 1h at room temperature. CYS-3 and bound genomic DNA that was bound to the beads was washed and then heated to 98^o^C for 10 min to release DNA fragments into solution. The KAPA library kit for Illumina sequencing was used to prepare the DAPseq libraries for sequencing from these DNA fragments. A final DAPseq DNA library was generated in the same conditions with no plasmid added to the TnT Master Mix as a negative control. A single DAPseq library was sequenced for each condition with paired end 150bp reads on an Illumina MiSeq.

Filtered reads were aligned to the *N. crassa* OR74A genome (v12) using Bowtie2 v2.3.2 (9). Peak calling was performed using MACS2 v2.1.1 (10) with *P* value cutoff at 0.001 and utilizing negative control library alignments. Dataset S2 gives the full list of peaks that were called by MACS2 and within 3kbp upstream of a translational start site. To reduce the frequency of identifying false positive CYS-3 DNA binding sites, the DAPseq data was then further filtered for binding sites within 3kbp upstream of a translated gene that was at least 2-fold differentially expressed between wild type and *cys-3^-^* cells during exposure to media lacking sulfur or containing 24μM sulfate and had an FPKM of at least 10 in any one of these conditions.

DAPseq data used in this study were deposited in the NCBI Sequence Read Archive (SRA). The CYS-3 DAPseq data is accessible through SRA accession number SRX3748478 and the negative control DAPseq data is accessible through SRA accession number SRX3748477. Processed DAPseq data are available in Dataset S2.

***DNA binding consensus motif generation***

Motif discovery was performed using MEME v5.1.1 (11). The input for MEME motif discovery was DAPseq binding peak sequences with a maximum motif width of 20bp, a minimum motif width of 6bp, any number of motif sites in the sequences, the classic objective function, and a 0^th^ order Markov model for sequences. Binding peak sequences were included in motif generation if they were within 3kbp upstream of a translated gene that was at least 2-fold differentially expressed between wild type and *cys-3^-^* cells during exposure to media lacking sulfur or containing 24μM sulfate and had an FPKM of at least 10 in any one of these conditions.

**REFERENCES**

1. McCluskey K, Wiest A, Plamann M. 2010. The Fungal Genetics Stock Center: a repository for 50 years of fungal genetics research. J Biosci 35:119-126.

2. Colot HV, Park G, Turner GE, Ringelberg C, Crew CM, Litvinkova L, Weiss RL, Borkovich KA, Dunlap JC. 2006. A high-throughput gene knockout procedure for *Neurospora* reveals functions for multiple transcription factors. Proc Natl Acad Sci U S A 103:10352-10357.

3. Vogel H. 1956. A convenient growth medium for *Neurospora* (medium N). Microbial Genetics Bulletin 13:42-43.

4. Wu VW, Thieme N, Huberman LB, Dietschmann A, Kowbel DJ, Lee J, Calhoun S, Singan VR, Lipzen A, Xiong Y, Monti R, Blow MJ, O'Malley RC, Grigoriev IV, Benz JP, Glass NL. 2020. The regulatory and transcriptional landscape associated with carbon utilization in a filamentous fungus. Proc Natl Acad Sci U S A 117:6003-6013.

5. Galagan JE, Calvo SE, Borkovich KA, Selker EU, Read ND, Jaffe D, FitzHugh W, Ma LJ, Smirnov S, Purcell S, Rehman B, Elkins T, Engels R, Wang S, Nielsen CB, Butler J, Endrizzi M, Qui D, Ianakiev P, Bell-Pedersen D, Nelson MA, Werner-Washburne M, Selitrennikoff CP, Kinsey JA, Braun EL, Zelter A, Schulte U, Kothe GO, Jedd G, Mewes W, Staben C, Marcotte E, Greenberg D, Roy A, Foley K, Naylor J, Stange-Thomann N, Barrett R, Gnerre S, Kamal M, Kamvysselis M, Mauceli E, Bielke C, Rudd S, Frishman D, Krystofova S, Rasmussen C, Metzenberg RL, Perkins DD, Kroken S, et al. 2003. The genome sequence of the filamentous fungus *Neurospora crassa*. Nature 422:859-868.

6. Kim D, Langmead B, Salzberg SL. 2015. HISAT: a fast spliced aligner with low memory requirements. Nat Methods 12:357-360.

7. Trapnell C, Roberts A, Goff L, Pertea G, Kim D, Kelley DR, Pimentel H, Salzberg SL, Rinn JL, Pachter L. 2012. Differential gene and transcript expression analysis of RNA-seq experiments with TopHat and Cufflinks. Nat Protoc 7:562-578.

8. O'Malley RC, Huang SC, Song L, Lewsey MG, Bartlett A, Nery JR, Galli M, Gallavotti A, Ecker JR. 2016. Cistrome and epicistrome features shape the regulatory DNA landscape. Cell 165:1280-1292.

9. Kim D, Pertea G, Trapnell C, Pimentel H, Kelley R, Salzberg SL. 2013. TopHat2: accurate alignment of transcriptomes in the presence of insertions, deletions and gene fusions. Genome Biol 14:R36.

10. Zhang Y, Liu T, Meyer CA, Eeckhoute J, Johnson DS, Bernstein BE, Nusbaum C, Myers RM, Brown M, Li W, Liu XS. 2008. Model-based analysis of ChIP-Seq (MACS). Genome Biol 9:R137.

11. Bailey TL, Boden M, Buske FA, Frith M, Grant CE, Clementi L, Ren J, Li WW, Noble WS. 2009. MEME SUITE: tools for motif discovery and searching. Nucleic Acids Res 37:W202-8.
