# Supplementary material for: Aspects of the Neurospora crassa Sulfur Starvation Response Are Revealed by Transcriptional Profiling and DNA Affinity Purification Sequencing
Source: mSphere. 2021 Sep 15;6(5):e00564-21. doi: 10.1128/mSphere.00564-21 (PMC8550094; doi:10.1128/mSphere.00564-21)
Supplement: TABLE S1 [file msphere.00564-21-st001.docx]

| Chemical | Amount (g) |
| --- | --- |
| Citric acid monohydrate | 5 |
| Zinc chloride | 2.37 |
| Iron(II) chloride tetrahydrate | 0.397 |
| Copper(II) chloride dihydrate | 0.171 |
| Manganese(II) chloride tetrahydrate | 0.059 |
| Dihydrogen borate | 0.05 |
| Sodium molybdate dihydrate | 0.05 |
